# Contrast-enhanced chest computed tomography in acute heart failure: a narrative review of imaging clues to precipitating factors and underlying cardiac disease

**DOI:** 10.12701/jyms.2026.43.59

**Published:** 2026-08-29

**Authors:** Charles S. White, Seung Min Yoo

**Affiliations:** 1 Department of Radiology, University of Maryland, Baltimore, MD, USA; 2 Department of Radiology, CHA University Bundang Medical Center, Seongnam, Korea

**Keywords:** Acute heart failure, Computed tomography, Enhancement

## Abstract

Acute heart failure (AHF) is a clinical syndrome characterized by the rapid onset or worsening of symptoms and signs resulting from structural or functional cardiac abnormalities and may present with systemic congestion, hypoperfusion, or cardiogenic shock. AHF can be divided into de novo AHF (i.e., new onset) and acute decompensated heart failure (i.e., rapid clinical deterioration in patients with a previous diagnosis of heart failure) and can be caused by various precipitating factors (e.g., acute myocardial infarction, nonadherence to heart failure medications, high salt intake, arrhythmia, and hypertensive crisis). AHF is often diagnosed based on the symptoms and signs of heart failure, supported by elevated serum levels of N-terminal pro-B-type natriuretic peptide and chest radiographic and echocardiographic abnormalities. Contrast-enhanced chest computed tomography (CT) is not routinely used for the diagnostic workup of AHF. However, CT is increasingly used in patients with indeterminate clinical presentations in the emergency department (ED) to discriminate AHF from multifocal pneumonia or acute respiratory distress syndrome. In this context, CT may be the initial imaging tool for patients with AHF who present to the ED. Owing to advances in CT technology, careful assessment of routine chest CT findings may help determine the specific etiology of AHF, especially new-onset AHF. This review provides important tips for identifying diagnostic clues of AHF based on CT findings.

## Introduction

Chronic heart failure (CHF) is a clinical condition characterized by the gradual onset of pulmonary symptoms (dyspnea and orthopnea) and/or systemic congestion (jugular vein distension and lower extremity edema) secondary to structural or functional cardiac abnormalities. The abbreviation HF (i.e., heart failure) is often used as a shortened term for CHF. Acute heart failure (AHF) is a clinical syndrome characterized by the rapid onset or worsening of symptoms and signs resulting from structural or functional cardiac abnormalities and may present with systemic congestion, hypoperfusion, or cardiogenic shock. AHF can be subdivided into de novo AHF (i.e., new onset) and acute decompensated HF (ADHF; i.e., rapid clinical deterioration in patients with a previous diagnosis of HF) and is caused by various precipitating factors (e.g., acute myocardial infarction [AMI], nonadherence to HF medications, high salt intake, arrhythmia, and hypertensive crisis) [1-4]. The requirement for hospital admission in patients with ADHF often leads to an unfavorable prognosis, with frequent repeat hospitalizations and a high post-discharge mortality rate [3]. In fact, one study indicated that the mortality rates during the index admission, 30 days post-discharge, and 1 year post-discharge were 10%, 6.5%, and 30%, respectively [5]. Compared with ADHF, de novo AHF generally has a better prognosis. The favorable outcomes in patients with de novo AHF may be associated with fewer comorbidities than in those with ADHF and more commonly with correctable triggering events (e.g., AMI or acute mitral regurgitation due to acute rupture of chordae tendineae) [3]. Additionally, the clinical presentation of de novo AHF may differ from that of ADHF. Patients with de novo AHF more commonly present with cardiogenic shock or acute cardiogenic pulmonary edema (ACPE) than those with ADHF (who present with acute pulmonary congestion alone or pulmonary edema) [3]. The diagnosis of AHF is often driven by clinical context and supported by laboratory, chest radiographic, and echocardiographic findings. AHF can also be classified as AHF with reduced ejection fraction (EF) (≤40%), AHF with mildly reduced EF (41%–49%), and AHF with preserved EF (≥50%) [6,7]. Contrast-enhanced chest computed tomography (CT) is not included in the routine diagnostic workup for AHF [8]. However, contrast-enhanced chest CT is increasingly used in patients with AHF and indeterminate clinical presentations in the emergency department (ED), and differentiation of acute respiratory distress syndrome (ARDS) or multifocal pneumonia from AHF may not be feasible based solely on the initial clinical assessment [9,10]. In this situation, CT can be a valuable option for differentiating AHF from other conditions with a similar presentation [9,10]. Moreover, with advancements in CT technology, careful assessment of chest CT findings may lead to the diagnosis of a specific AHF etiology [11,12]. In contrast to previous review papers that only dealt with each entity resulting in AHF (e.g., acute coronary syndrome), this review provides important tips for distinguishing AHF from ARDS, and it provides representative illustrations to demonstrate diagnostic clues for AHF with reduced systolic function on routine chest CT.

## Typical computed tomography findings of acute heart failure and mimics

The presence of cardiac chamber enlargement (left atrium and/or left ventricle), bilateral pleural effusions, central predominance of ground-glass opacity (GGO), and interlobular septal thickening on CT may suggest ACPE due to AHF rather than ARDS, the latter of which typically demonstrates peripheral predominance of GGO and more widespread consolidation without interlobular septal thickening (Fig. 1) [9,10]. However, there may be a significant overlap in CT findings between ACPE and ARDS. Thus, it should be emphasized that a combination of CT findings, including cardiac chamber enlargement, smooth septal and peribronchovascular interstitial thickening, pleural effusions, and distribution of pulmonary opacities, should be interpreted together with clinical, biomarker, and echocardiographic findings (Table 1).

## Computed tomography identification of acute triggering factors

AHF (both de novo AHF and ADHF) may result from various triggering events denoted by the CHAMPIT acronym (i.e., acute coronary syndrome, hypertensive emergency, arrhythmias, mechanical causes, pulmonary embolism, infection, tamponade) [3]. The immediate identification of triggering events may lead to timely and appropriate treatment for patients with AHF [3]. Among the triggering events that lead to AHF, CT can identify AMI, mechanical causes, pulmonary embolism, infection, and cardiac tamponade.

### 1. Acute myocardial infarction

AMI may result from rupture of vulnerable plaques. AMI encompasses ST-segment elevation AMI and non-ST-segment elevation myocardial infarction. According to the fourth universal definition, AMI is defined as the presence of acute myocardial injury detected by abnormal cardiac troponin levels (above the 99th percentile upper reference limit) with a rise and/or fall coupled with clinical evidence of acute myocardial ischemia [13]. Although high-sensitivity cardiac troponin is now the recommended standard for contemporary diagnostic pathways, conventional cardiac troponin is still used in the ED. Conventional cardiac troponin levels may remain normal for up to 6 hours after an AMI episode. Furthermore, the initial resting electrocardiogram (ECG) may be normal or nonspecific even in patients who ultimately are found to have AMI. Thus, CT abnormalities may indicate the presence of AMI in patients in the ED with nonspecific acute chest pain and/or dyspnea unaccompanied by initial ischemic ECG changes or troponin elevation [14]. Typical CT findings of AMI on non-ECG-gated chest CT include subendocardial or transmural hypoattenuation involving a specific coronary artery territory. In contrast to cardiac motion artifacts, true AMI is characterized by minimal or no motion artifacts, homogeneous low attenuation in contrast to the higher attenuation of the normal myocardium, and conformation to a specific coronary artery territory (Fig. 2) [14-16]. However, it should be noted that myocardial hypoattenuation on non-gated CT should be presented as an important clue that warrants urgent clinical, laboratory, and cardiac evaluation, not as an independent diagnostic criterion for AMI. Notably, regarding the low myocardial attenuation at the left ventricular (LV) base, it is important to recognize potential false-positive findings caused by beam-hardening artifacts. Specifically, the polyenergetic nature of the X-ray beam can cause low attenuation in the myocardium adjacent to the dense contrast material within the LV blood pool, particularly along the posterobasal wall, mimicking a true perfusion defect [17].

### 2. Mechanical causes

The most common cause of acute mitral valve regurgitation is AMI (specifically papillary muscle rupture in patients with AMI involving the inferolateral wall). Compared to the anterolateral papillary muscle, the posteromedial papillary muscle is more vulnerable to ischemic insult owing to its single vascular supply, potentially leading to papillary muscle necrosis and resultant acute mitral regurgitation [18]. Acute mitral regurgitation may occur less commonly due to acute chordal rupture, resulting in acute mitral valve prolapse and de novo AHF [19]. In one study, the asymmetric double-line sign on contrast-enhanced chest CT was defined as the presence of a linear structure not located in the plane traversing the mitral annulus. In that study, the asymmetric double-line sign predicted mitral valve prolapse with a sensitivity, specificity, negative predictive value, and positive predictive value of 59% (38/65), 99% (64/65), 70% (64/91), and 97% (38/39), respectively [20]. Thus, the presence of an asymmetric double-line sign on contrast-enhanced chest CT may indicate mitral valve prolapse (Fig. 3).

Another mechanical culprit lesion that causes de novo AHF is infective endocarditis. Non-ECG-gated contrast-enhanced chest CT is not a standard imaging tool for diagnosing infective endocarditis. However, contrast-enhanced chest CT may be used as an initial test in patients with infective endocarditis who present with nonspecific acute dyspnea and/or fever. One study indicated that the presence of an indirect oscillation sign on contrast-enhanced chest CT is suggestive of movable vegetation on echocardiography with low sensitivity (29.6%, 8/27) but high specificity (100%, 35/35) and positive predictive value (100%, 8/8) [21]. In that study, the indirect oscillation sign (Fig. 4) was defined as a combination of elongated nodular valve thickening and abnormal orientation relative to the normal valve with a blind end on contrast-enhanced chest CT [21]. In the presence of this configuration, the elongated nodular thickening attached to the normal aortic or mitral valve should be mobile when the heart contracts (i.e., an indirect oscillation sign). In patients with infective endocarditis, perforation of the valve leaflet may occur due to severe inflammation or necrosis, resulting in acute aortic or mitral regurgitation and de novo AHF. The Z-axis coverage of chest CT often includes the upper portions of the spleen and both kidneys. The simultaneous presence of splenic or renal infarction caused by an embolic shower of vegetations and the indirect oscillation sign on contrast-enhanced chest CT may indicate infective endocarditis [21].

### 3. Pulmonary embolism

Massive pulmonary embolism (high-risk pulmonary embolism) is a life-threatening condition caused by occlusion of the main pulmonary arteries due to multiple pulmonary emboli accompanied by hemodynamic instability (defined as systolic blood pressure <90 mmHg or shock) [22]. A high-risk pulmonary embolism (Fig. 5) may hinder the efficient transit of blood from the right to the left circulation, leading to reduced LV output and tissue hypoperfusion. Immediate thrombolytic therapy may be lifesaving in the absence of contraindications.

### 4. Infection

Infections (such as those causing pneumonia or septic shock) may impose an additional burden on the heart in patients with preexisting CHF, leading to ADHF [3]. Thus, the possibility of superimposed pneumonia should be carefully evaluated in patients with AHF by using CT. In contrast to the strong enhancement in a purely atelectatic lung on contrast-enhanced chest CT, a pneumonic focus often demonstrates inhomogeneous low attenuation owing to the accumulation of exudative fluid within the alveoli (Fig. 6).

### 5. Tamponade

Cardiac tamponade is a medical emergency characterized by diastolic compression of the right ventricle or atrium due to rapid accumulation of pericardial fluid or blood. Causes of cardiac tamponade include chest trauma, iatrogenic etiology, malignancy, and pericarditis [23]. It is rare for radiologists to observe cardiac tamponade on contrast-enhanced chest CT, mainly because hemodynamic instability renders the patient too unstable to undergo CT. Although rare, the typical CT findings of cardiac tamponade include compression of the right atrium or ventricle by pericardial fluid collection (Fig. 7) [24].

## Computed tomography identification of underlying cardiac disease

CT has the potential to identify preexisting cardiac dysfunction (e.g., ischemic cardiomyopathy, valvular cardiomyopathy, and dilated cardiomyopathy [DCM] phenotypes).

### 1. Ischemic cardiomyopathy

One study indicated that visual assessment of coronary calcification on contrast-enhanced chest CT showed good correlation with the coronary artery calcium score on ECG-gated cardiac CT [25]. In that study, severe coronary calcification was defined as extensive or diffuse calcification throughout the coronary arteries as observed on contrast-enhanced chest CT. Ho et al. [26] indicated that a higher coronary artery calcium score was associated with a greater prevalence of significant coronary artery stenosis on cardiac CT independent of traditional risk factors. However, extensive coronary artery calcification indicates severe atherosclerotic burden but does not establish obstructive coronary disease or ischemic cardiomyopathy. In this regard, the presence of extensive coronary calcifications on chest CT scans in patients with AHF should only be interpreted as a clue to an ischemic phenotype (Fig. 8).

In addition to severe coronary artery calcification, the identification of chronic myocardial infarction on chest CT suggests preexisting ischemic cardiomyopathy in patients presenting with ADHF. The typical CT features of chronic myocardial infarction include fatty metaplasia and/or myocardial thinning in a subendocardial or transmural distribution along a specific coronary artery territory or the presence of aneurysm formation (Fig. 9) [27].

### 2. Aortic valve disease

#### 1) Severe aortic stenosis

The most common causes of aortic valve stenosis are degenerative changes, although a bicuspid aortic valve is a major cause of severe aortic valve stenosis in younger patients. Previous studies have indicated that visual assessment of aortic valve calcification (Fig. 10) on contrast-enhanced chest CT is associated with the severity of aortic valve stenosis on echocardiography [28-31]. The presence of severe aortic valve calcification on CT in patients presenting with ADHF often suggests severe aortic stenosis as a cause of the underlying cardiac dysfunction.

#### 2) Severe aortic regurgitation

Aortic regurgitation cannot be directly assessed on non-ECG-gated CT. However, an increase in the diameter of the sinus of Valsalva (>40 mm) (Fig. 11A) suggests aortic regurgitation secondary to incomplete coaptation of the aortic valve cusps [32]. Although sinus of Valsalva dilatation may contribute to aortic regurgitation, it should be noted that the CT findings do not establish its presence or severity. Rarely, incomplete coaptation of the aortic valve cusps (Fig. 11B) can be demonstrated on a diastolic phase image of contrast-enhanced chest CT, although this is more commonly observed on ECG-gated cardiac CT [33].

### 3. Mitral valve disease

#### 1) Severe mitral regurgitation

As previously described, mitral valve prolapse (the asymmetric double-line sign) or infective endocarditis (the indirect oscillation sign that may be identified on contrast-enhanced chest CT) are underlying cardiac abnormalities leading to ADHF [19,20]. However, the use of asymmetric double-line and indirect oscillation signs is supported by small studies, and these indicators have limited sensitivity. Their high specificity is clinically useful; however, the absence of these CT signs cannot exclude the possibility of disease.

#### 2) Severe mitral stenosis

Although rare in developed countries, rheumatic mitral stenosis can also cause ADHF. In contrast to mitral annular calcification, calcification and thickening of the mitral valve are typical CT findings in rheumatic mitral stenosis (Fig. 12) [34,35].

### 4. Dilated cardiomyopathy

DCM is a leading cause of cardiac transplantation, particularly in younger patients. DCM is defined as LV dilatation with systolic dysfunction in the absence of other conditions with phenotypic overlap (Fig. 13) [36]. DCM should be diagnosed after extensive workup and exclusion of other conditions that result in the same phenotype (e.g., toxins, infections, metabolic conditions, autoimmunity, pregnancy, tachyarrhythmia, and neuromuscular disease). The identification of the specific etiology leading to DCM is important because targeted treatment specific to that etiology may result in better outcomes [36]. In one study, DCM was defined as LV enlargement (>55 mm in end-diastole) with decreased LV systolic function (EF <40%) and the absence of significant obstructive coronary artery disease on ECG-gated cardiac CT. In addition, myocardial thinning (<12 mm) in patients with DCM may occur at the end-stage of the disease (i.e., LV myocardial thinning is only observed in patients with full-blown DCM) [37]. Thus, LV dilatation and apparent wall thinning in a randomly acquired cardiac phase may suggest a DCM phenotype but cannot establish a diagnosis of DCM. Ischemic cardiomyopathy should be excluded before diagnosing DCM because the phenotypes of DCM and ischemic cardiomyopathy may be identical, except for the presence of multivessel disease on coronary angiography in the latter [38]. It is important to understand that DCM is the final response of the myocardium to a variety of cardiac insults, including ischemic, inflammatory, genetic, and toxic [36]. The typical CT features indicating diagnostic clues for AHF are summarized in Table 2.

## Technical limitations and clinical integration

The interpretation of cardiac findings on routine chest CT is limited by the absence of ECG synchronization, random cardiac phase, cardiac and valvular motion, and variability in contrast timing. Beam hardening may mimic myocardial hypoattenuation, and the chamber dimensions and wall thickness measured on non-gated acquisition may be inaccurate. Small vegetations and subtle leaflet abnormalities may be missed because of the limited temporal and spatial resolution. Diagnostic visibility also differs between CT pulmonary angiography, arterial-phase chest CT, and later-phase examinations. Accordingly, suspected abnormalities should be integrated with symptoms, ECG, biomarkers, and prior imaging, and confirmed with echocardiography, coronary angiography, or cardiac magnetic resonance imaging when appropriate. Table 2 summarizes the principal CT clues, limitations, and recommended confirmatory tests.

## Conclusion

The presence of cardiac chamber enlargement (left atrial and/or LV), bilateral pleural effusions, central predominance of GGO, and interlobular septal thickening on CT favors ACPE due to AHF rather than ARDS. Among the triggering factors leading to AHF, CT has the potential to identify the presence of AMI, mechanical causes, pulmonary embolism, infection, and cardiac tamponade. In addition, CT has the potential to identify preexisting cardiac dysfunction (e.g., ischemic, valvular, and DCM phenotypes). Thus, cardiac findings on routine chest CT provide clues that can direct prompt confirmatory testing and management, rather than establish the final etiology of AHF in isolation.

## Figures and Tables

**Fig. 1.**
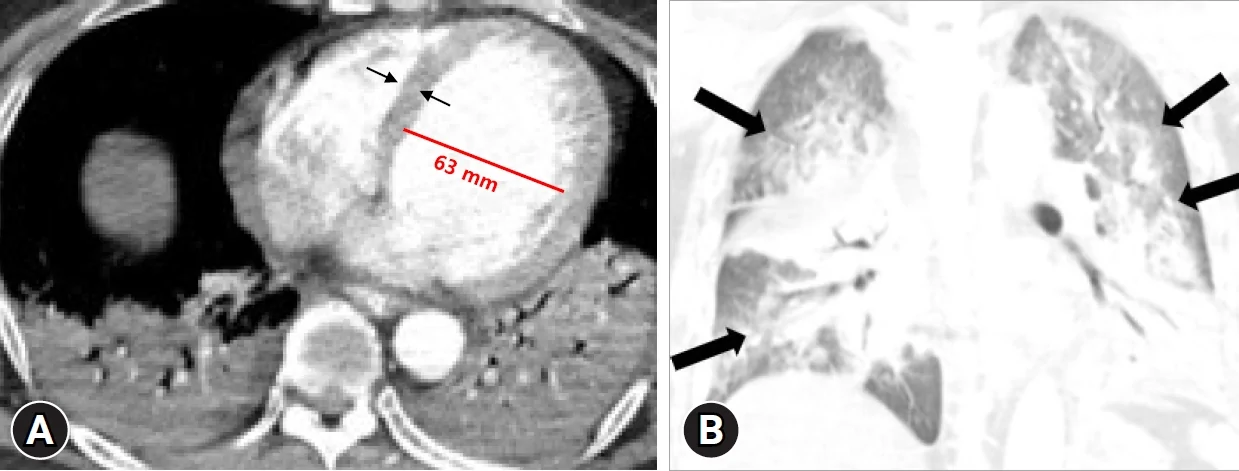
Case of cardiogenic pulmonary edema in a 57-year-old man presenting with acute dyspnea. The serum N-terminal pro-B-type natriuretic peptide was elevated (525 pg/mL; normal, ≤263 pg/mL). (A) The left ventricular diameter is 63 mm, indicating marked enlargement. The left ventricular wall (arrows) shows no significant thinning or hypertrophy. (B) Note the central predominance of ground-glass attenuations and consolidations in both lungs (arrows) on this coronal computed tomography image.

**Fig. 2.**
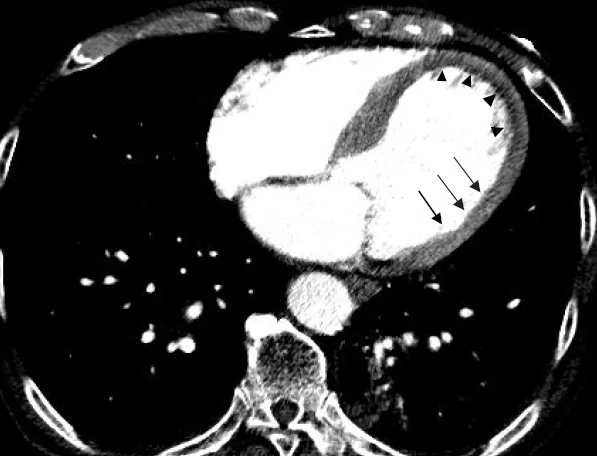
Case of acute myocardial infarction in a 76-year-old man presenting to the emergency department with new-onset acute heart failure. In contrast to the normal higher attenuation (arrows) in the lateral wall of the left ventricle, there is subendocardial hypoattenuation (arrowheads) along the left anterior descending coronary artery territory (around the left ventricular apex on this axial computed tomography image). Emergent coronary angiography confirms total occlusion of the proximal left anterior descending coronary artery.

**Fig. 3.**
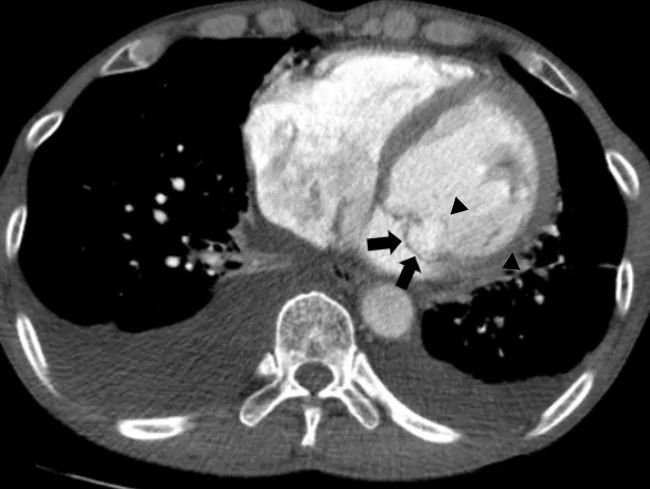
Case of mitral valve prolapse in a 57-year-old man presenting with acute dyspnea. Note the asymmetric double-line sign (arrows and arrowhead) of the mitral valve cusp suggesting the presence of mitral valve prolapse. In this case, sudden tearing of mitral chordae tendineae has resulted in acute cardiogenic pulmonary edema (i.e., de novo acute heart failure) due to acute mitral regurgitation. Computed tomography may be the initial diagnostic test leading to suspicion of mitral valve prolapse. Emergent mitral valve repair has been performed in this patient.

**Fig. 4.**
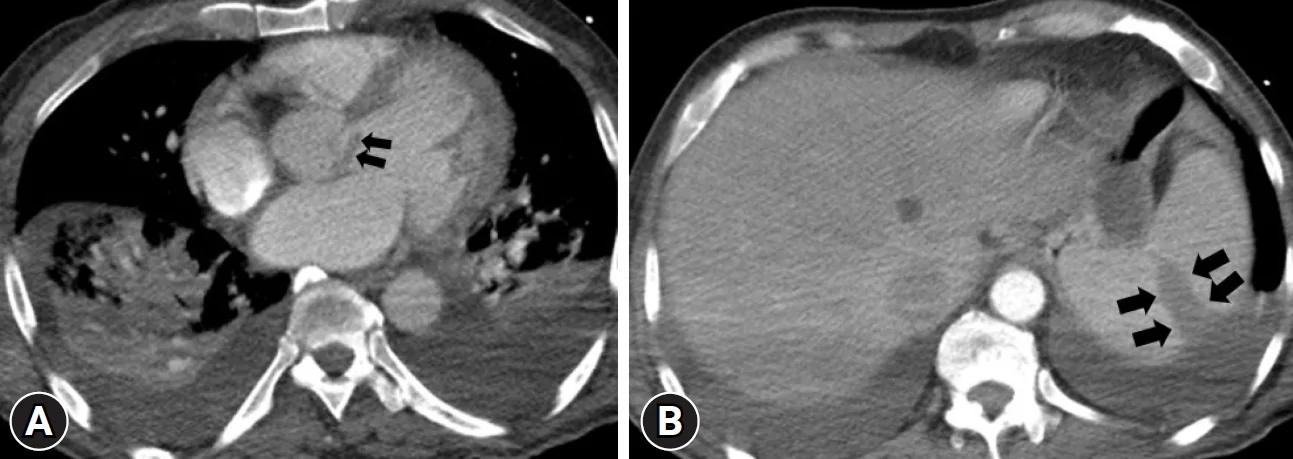
Case of infective endocarditis in a 62-year-old man presenting with acute dyspnea. (A) Note nodular thickening (arrows) attached to the aortic valve on axial computed tomography (CT) suggesting the presence of a vegetation. The elongated nodular thickening is not a normal component of an aortic valve cusp because it has a free end directed into the left ventricle. This is an important finding that can differentiate a vegetation from a normal aortic valve cusp. (B) Note the wedge-shaped splenic infarction on the axial CT image (arrows). The combination of splenic infarction and nodular thickening on the aortic valve may indicate infective endocarditis based on chest CT.

**Fig. 5.**
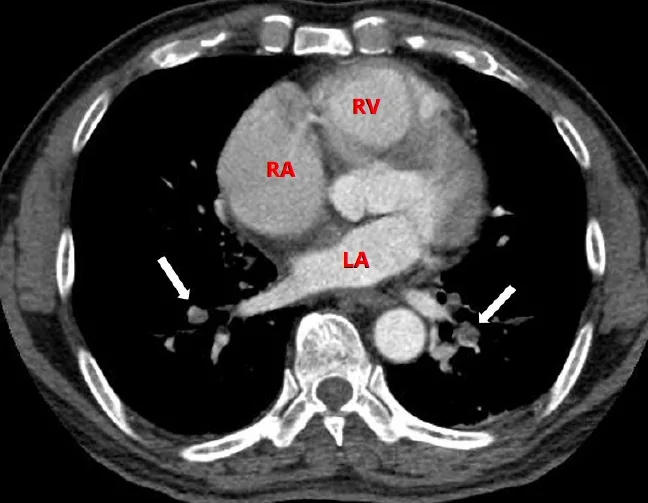
Case of high-risk pulmonary embolism in a 63-year-old man. Note multiple pulmonary emboli in both lower lobes (arrows) and marked right ventricular and atrial enlargement. RV, right ventricle; RA, right atrium; LA, left atrium.

**Fig. 6.**
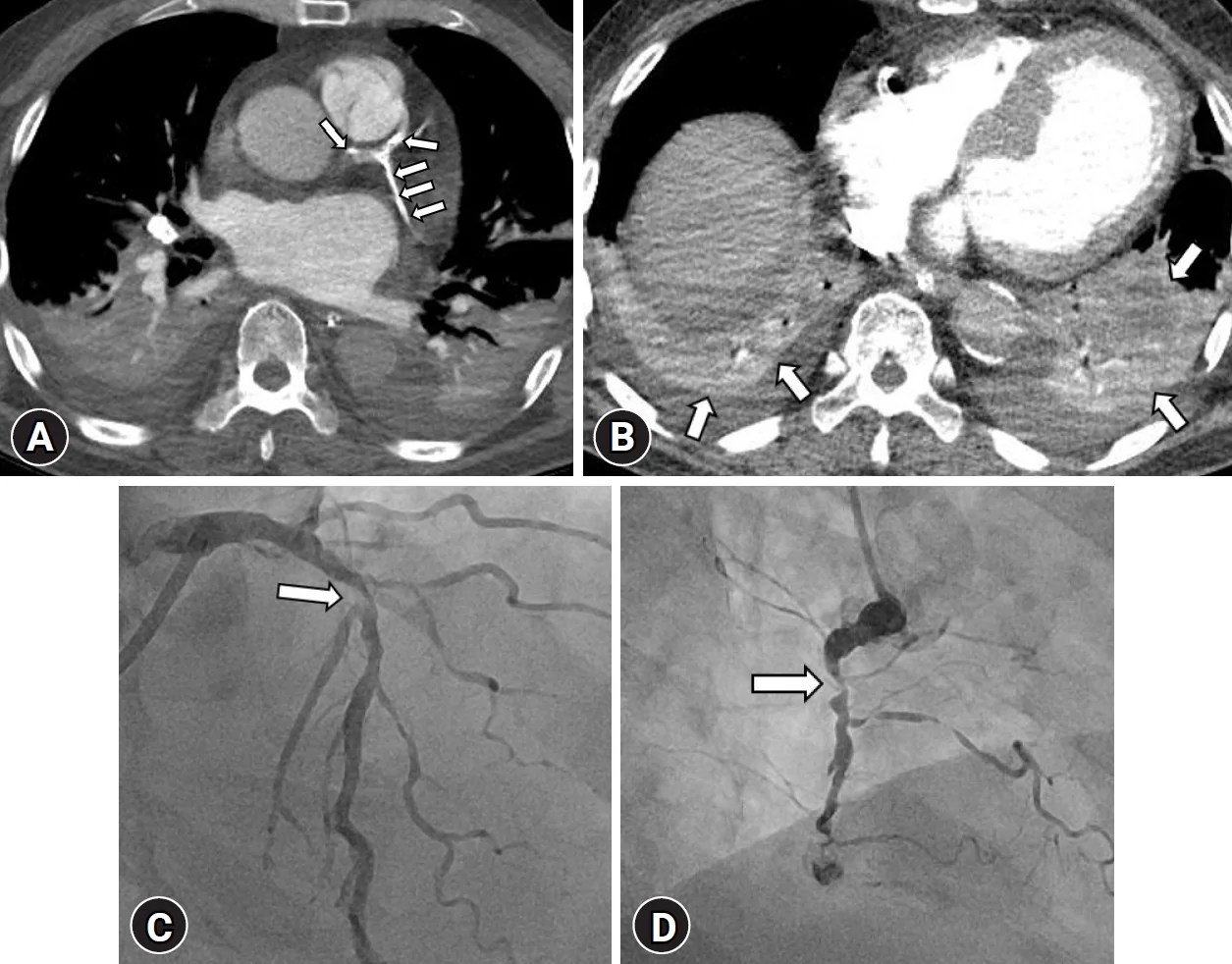
Case of acute decompensated heart failure precipitated by pneumonia with preexisting ischemic cardiomyopathy in a 75-year-old man. (A) Note multiple severe coronary artery calcifications (arrows) in the left anterior descending and left circumflex coronary arteries, suggesting the possibility of preexisting ischemic cardiomyopathy. (B) There are moderate bilateral pleural effusions, left ventricular dilatation, and consolidations in both lower lobes. As opposed to typical atelectasis, attenuation of the consolidations in both lower lobes is inhomogeneous (arrows) on the enhanced computed tomography image, suggesting pneumonic foci. Subsequent coronary angiography confirms critical stenoses (arrows) in the proximal left anterior descending (C) and right coronary (D) arteries.

**Fig. 7.**
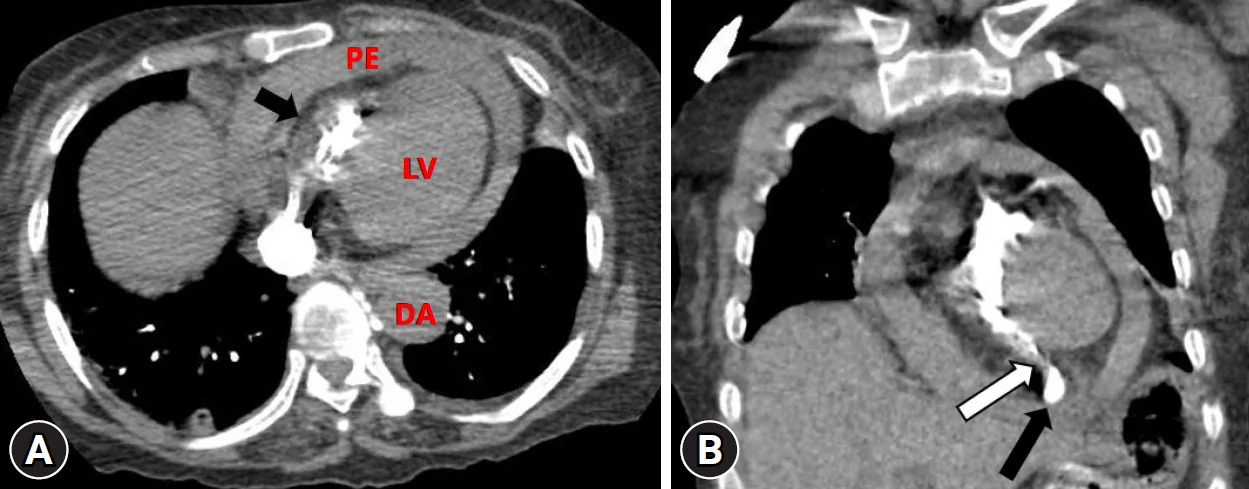
Case of tamponade due to right ventricular rupture in a 75-year-old woman with chronic renal failure. The patient has a long-term indwelling catheter in the superior vena cava. She presents to the emergency department with cardiogenic shock. (A) On contrast-enhanced chest computed tomography (CT), there is a moderate pericardial effusion (PE) with high attenuation (>40 Hounsfield units), suggesting hemopericardium. Note the small right ventricle due to compression by the hemopericardium and poor enhancement in the left ventricle (LV) and descending aorta (DA), suggesting cardiogenic shock. (B) On a coronal CT image, there is contrast leakage (black arrow) from the right ventricular apex (white arrow), suggesting perforation. Emergent surgery confirms the CT findings.

**Fig. 8.**
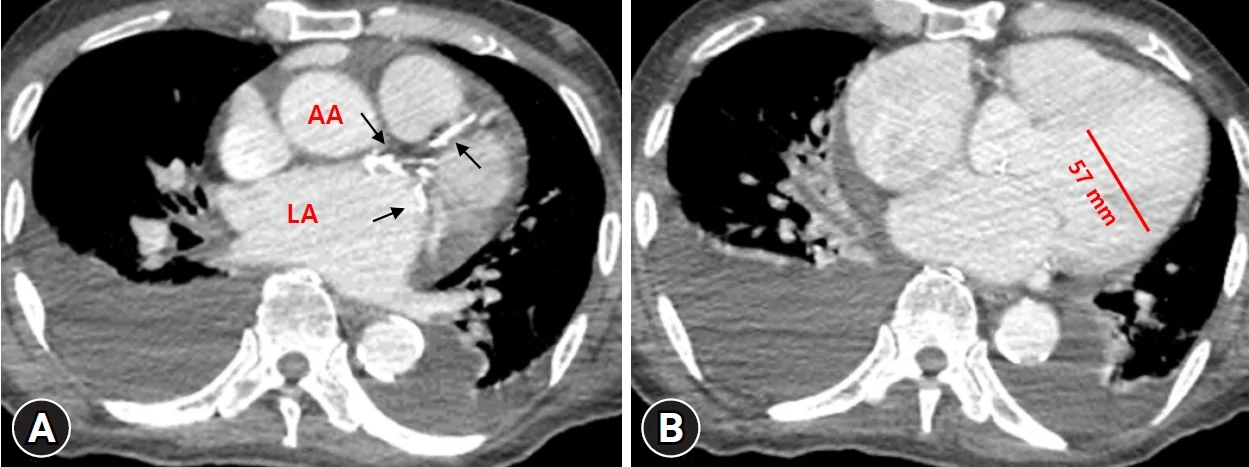
A 75-year-old man with ischemic cardiomyopathy as an underlying cause of acute decompensated heart failure. (A) Contrast-enhanced chest computed tomography (CT) demonstrates severe coronary artery calcification (arrows), moderate bilateral pleural effusions, and left atrial and ventricular enlargement. Note marked increase in the diameter of the left atrium (LA) compared to that of the ascending aorta (AA). (B) The left ventricular diameter measures 57 mm, indicating enlargement. This constellation of CT findings suggests ischemic cardiomyopathy as a cause of preexisting heart failure in this patient.

**Fig. 9.**
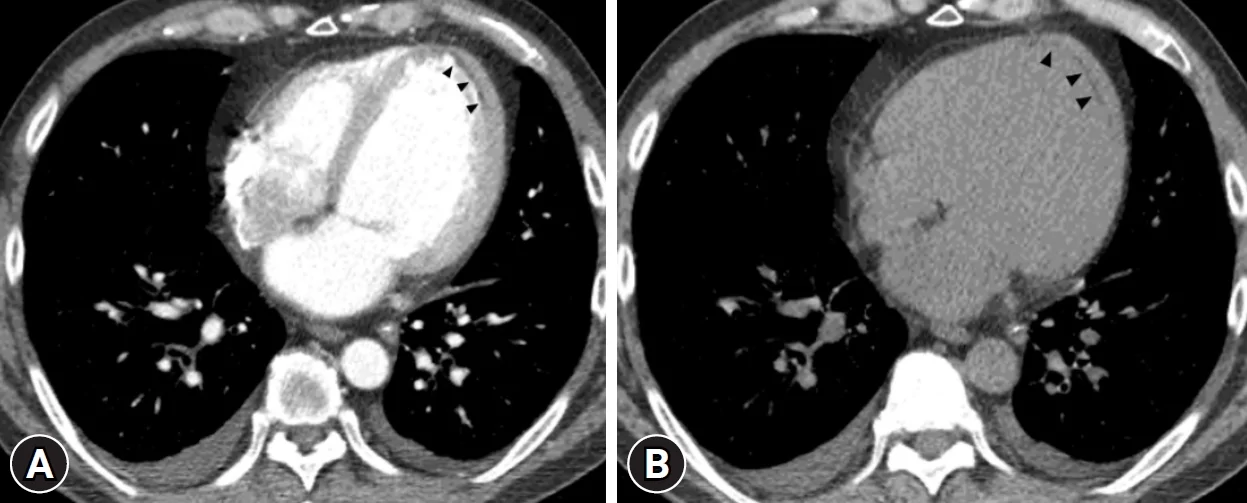
Case of chronic myocardial infarction in a 49-year-old man. (A) Note subendocardial hypoattenuation with myocardial thinning near the left ventricular apex on an enhanced axial computed tomography (CT) image (arrowheads). (B) There is fatty metaplasia in the same area (arrowheads) on an axial precontrast CT image. The above CT findings are consistent with chronic myocardial infarction in a left anterior descending coronary artery distribution.

**Fig. 10.**
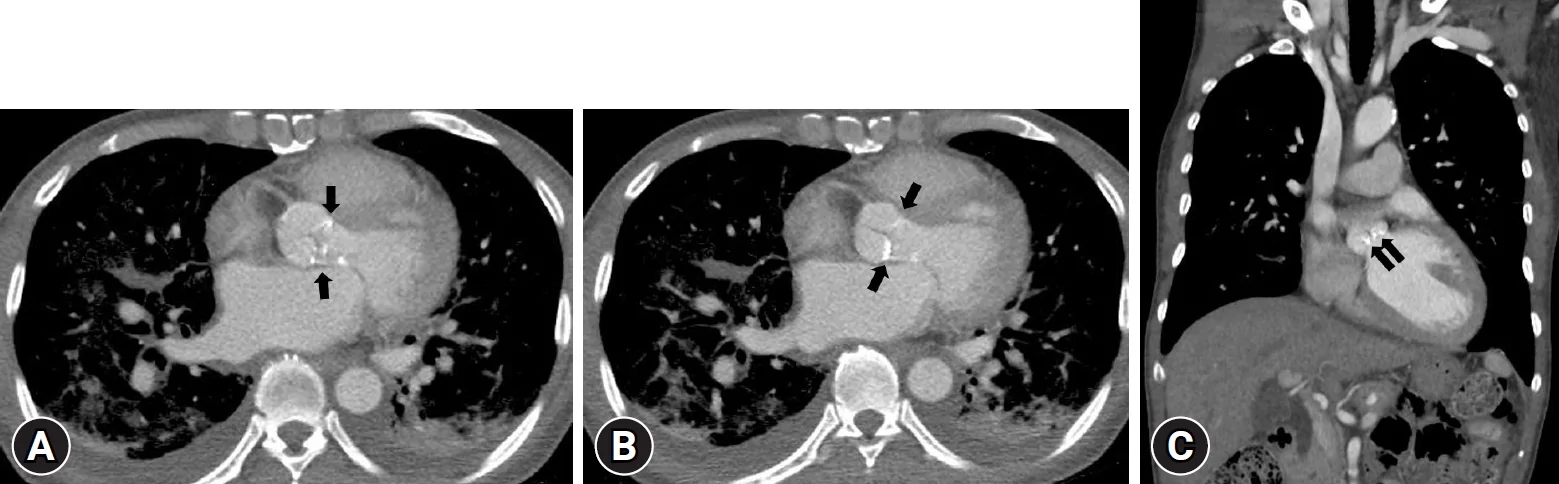
Case of severe aortic stenosis in a 76-year-old woman. (A, B) Note multiple aortic valve calcifications (arrows), bilateral pleural effusions, and marked left atrial enlargement. (C) There are multiple aortic valve calcifications (arrows) on the coronal computed tomography image.

**Fig. 11.**
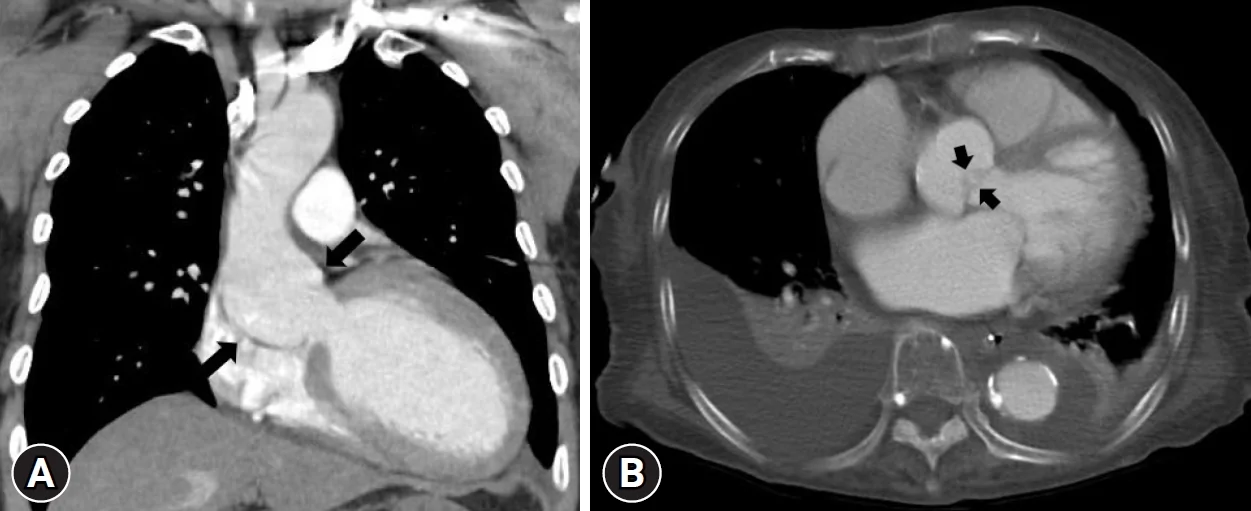
Indirect computed tomography signs suggesting the presence of aortic regurgitation. (A) Case of sinus of Valsalva dilatation with severe aortic regurgitation in a 59-year-old man. The sinus of Valsalva diameter (between the arrows) is 49 mm on the coronal view in this patient. (B) Case of severe aortic regurgitation in an 85-year-old woman. Note the relatively large gap (between the arrows) in the aortic valve indicating incomplete coaptation of the aortic valve on a diastolic phase computed tomography image. Severe aortic regurgitation is confirmed by subsequent echocardiography.

**Fig. 12.**
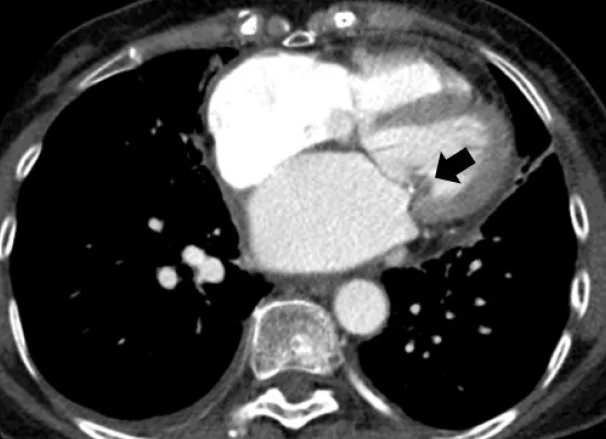
Case of rheumatic mitral stenosis in a 64-year-old woman presenting with dyspnea. Note calcification and thickening (arrow) of the mitral valve proper with enlarged left atrium on chest computed tomography, consistent with rheumatic mitral stenosis.

**Fig. 13.**
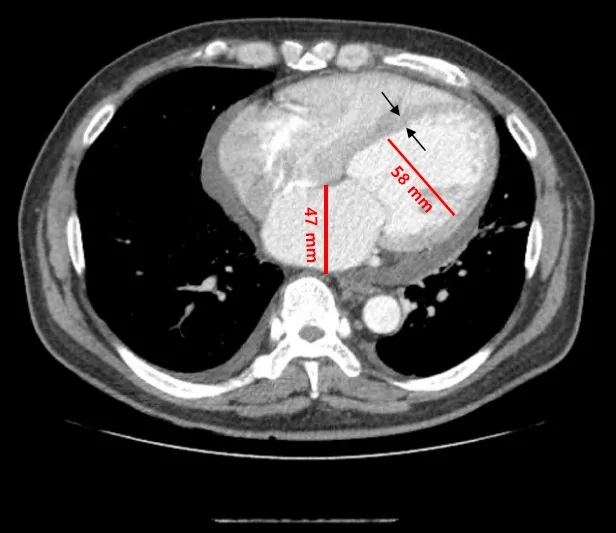
Case of dilated cardiomyopathy phenotype in a 53-year-old man. The patient is a long-standing alcohol user. Note the marked dilation of the left ventricle (58 mm) and left atrium (47 mm) with thinning (8 mm between the arrows) of the left ventricle. The combination of these computed tomography findings suggests dilated cardiomyopathy as a cause of acute decompensated heart failure.

**Table 1.** Computed tomography features that may support cardiogenic pulmonary edema over major pulmonary mimics

Feature	Cardiogenic pulmonary edema	ARDS or multifocal pneumonia
Cardiac chambers	Left atrial and/or left ventricular enlargement may be present	Usually no primary left-sided chamber enlargement
Ground-glass opacity	Often central, perihilar, or dependent	Often patchy or peripheral, but substantial overlap occurs
Septal/interstitial change	Smooth interlobular septal and peribronchovascular interstitial thickening	May occur, particularly in mixed or later-stage disease
Pleural effusion	Common and often bilateral	Less typical in uncomplicated ARDS; may occur with infection or volume overload
Consolidation	May accompany edema and is often dependent	Often more extensive or heterogeneous; focality may support pneumonia
Interpretation	Use the combination of cardiac and pulmonary findings	Integrate distribution with clinical data, biomarkers, and echocardiography

ARDS, acute respiratory distress syndrome.

**Table 2.** CT clues to precipitating factors and underlying cardiac disease in acute heart failure

Condition	Principal CT clue	Main limitation	Recommended confirmation
Acute MI	Territorial subendocardial or transmural hypoattenuation	Motion or beam-hardening may mimic a defect	Serial hs-cTn, ECG, echocardiography, coronary evaluation
Acute MR/MVP	Abnormal leaflet configuration or asymmetric double-line sign	Low sensitivity and limited temporal resolution	Urgent echocardiography
Infective endocarditis	Valve lesion with a free end; concomitant splenic or renal infarction	Small vegetations may be missed	Echocardiography; microbiologic evaluation
Pulmonary embolism	Pulmonary arterial filling defect with right-heart strain	Hemodynamic severity is not determined by clot burden alone	Clinical risk assessment and echocardiography
Pneumonia/infection	Focal or heterogeneous low-attenuation consolidation	Overlap with edema and atelectasis	Clinical, laboratory, and microbiologic correlation
Cardiac tamponade	Pericardial fluid or blood with right atrial or ventricular compression	Tamponade is a hemodynamic diagnosis	Urgent echocardiography and clinical assessment
Chronic ischemic phenotype	Extensive coronary calcification or chronic infarct scar	Calcification alone does not establish ischemia or cardiomyopathy	Coronary evaluation; echocardiography or CMR
Aortic stenosis	Marked aortic valve calcification	Non-gated CT does not grade stenosis	Echocardiography
Aortic regurgitation	Root dilatation or suspected incomplete cusp coaptation	Random phase; CT does not quantify regurgitation	Echocardiography or CMR
Rheumatic mitral stenosis	Leaflet thickening or calcification with left atrial enlargement	Mitral annular calcification may confound assessment	Echocardiography
Dilated cardiomyopathy phenotype	Marked left ventricular dilatation with apparent wall thinning	Phase-dependent measurements; etiology cannot be assigned	Echocardiography or CMR; coronary evaluation when indicated

CT, computed tomography; MI, myocardial infarction; hs-cTn, high-sensitivity cardiac troponin; ECG, electrocardiogram; MR, mitral regurgitation; MVP, mitral valve prolapse; CMR, cardiac magnetic resonance.
